# ULTRASONOGRAPHIC MARKERS OF CARDIOVASCULAR DISEASE RISK IN OBESE CHILDREN

**DOI:** 10.1590/1984-0462/;2018;36;2;00016

**Published:** 2018-03-29

**Authors:** Karla Cristina Malta Costa, Luiz Antonio Del Ciampo, Patrícia Silveira Silva, Jailson Costa Lima, Wellington de Paula Martins, Carlos Alberto Nogueira-de-Almeida

**Affiliations:** a Faculdade de Medicina de Ribeirão Preto, Universidade de São Paulo, Ribeirão Preto, SP, Brasil.

**Keywords:** Child, Obesity, Cardiovascular disease, Endothelium, Ultrasonography, Criança, Obesidade, Doenças cardiovasculares, Endotélio, Ultrassonografia

## Abstract

**Objective:**

To evaluate whether the obesity alters ultrasonographical markers of metabolic and cardiovascular disease risk in children.

**Methods:**

A cross-sectional study evaluated 80 children aged between 6 and 10 years, comparing 40 obese with 40 normal children. The following parameters were assessed: weight; height; body mass index; arterial blood pressure; body fat; basal metabolic rate; HDL-cholesterol, LDL-cholesterol and total cholesterol; fasting insulin and glucose; quantitative insulin sensitivity check index (QUICKI); homeostasis model of assessment - insulin resistance (HOMA-IR); basal diameter of the brachial artery; brachial artery flow mediated dilation (FMD) and of pulsatility index change (PI-C).

**Results:**

Significant differences were observed between obese vs. non-obese children: systolic blood pressure (97.7±8.4 vs. 89.0±5.8 mmHg; p<0.01), diastolic blood pressure (64.3±7.9 vs. 52.9±5.1 mmHg; p<0.01), proportion of body fat (45.1±5.9 vs. 21.3±6.0%; p<0.01), basal metabolic rate (1216.1±102.1 vs. 1072.9±66.4 Kcal; p<0.01), total cholesterol (164.7±25.2 vs. 153.4±15.8 mg/dL; p=0.03), fasting insulin (7.1±5.2 vs. 2.8±1.8 pIU/mL; p<0.01), HOMA-IR (1.5±1.1 vs. 0.6±0.4; p<0.01), basal diameter of the brachial artery (2.5±0.3 vs. 2.1±0.3 mm; p<0.01); PI-C (-15.5±27.2 vs. -31.9±15.5%; p<0.01), decreased QUICKI (0.4±0.05 vs. 0.4±0.03; p<0.01), and FMD (6.6±3.2 vs. 15.6±7.3%; p<0.01).

**Conclusions:**

Obesity worsens ultrasonographical and laboratorial markers of metabolic and cardiovascular disease risk in children.

## RESUMO

**Objetivo:**

Avaliar se a obesidade altera os marcadores ultrassonográficos de risco metabólico e cardiovascular em crianças.

**Métodos:**

Estudo transversal com 80 crianças entre 6 e 10 anos, comparando 40 crianças obesas com 40 crianças normais. Foram avaliados os seguintes parâmetros: peso; altura; índice de massa corporal; pressão arterial; massa gorda; taxa metabólica basal; HDL-colesterol, LDL-colesterol e colesterol total; insulina de jejum e glicose; índice quantitativo de verificação da sensibilidade à insulina (QUICKI); Homeostase Modell Assessment (HOMA-IR); Diâmetro basal da artéria braquial; Dilatação mediada pelo fluxo da artéria braquial (FMD) e variação do índice de pulsatilidade (PI-C).

**Resultados:**

Entre obesos e não obesos, observaram-se diferenças significativas na pressão arterial sistólica (97,7±8,4 *vs*. 89,0±5,8 mmHg; p<0,01), pressão arterial diastólica (64,3±7,9 *vs*. 52,9±5.1 mmHg; p<0,01), gordura corporal (45,1±5,9 *vs*. 21,3±6,0%; p<0,01), taxa metabólica basal (1216,1±102,1 *vs*. 1072,9±66,4 Kcal; p<0,01), colesterol (164,7±25,2 *vs*. 153,4±15,8 mg/dL; p=0,03), insulina de jejum (7,1±5,2 *vs*. 2,8±1,8 pIU/mL; p<0,01), HOMA-IR (1,5±1,1 *vs*. 0,6±0,4; p<0.01), diâmetro basal da artéria braquial (2,5±0,3 *vs*. 2,1±0,3 mm; p<0,01); PI-C (-15,5±27,2 *vs*. -31,9±15,5%; p<0,01), redução de QUICKI (0,4±0,05 *vs*. 0,4±0,03; p<0,01) e FMD (6,6±3,2 *vs*. 15,6±7,3%; p<0,01).

**Conclusões:**

A obesidade piora os marcadores ultrassonográficos e laboratoriais de risco metabólico e cardiovascular em crianças.

## INTRODUCTION

Obesity in childhood is an important risk factor to cardiovascular disease (CVD), dyslipidemia, impaired glucose tolerance, hypertension, adult obesity and premature mortality.^1^ ^,^ ^2^ In the last decades, the prevalence of obesity has markedly increased: about 10% of school-aged children worldwide are overweight; inwestern countries, approximately 35% are overweight, and about one fourth of those children are obese.^3^ ^,^ ^4^ Thus, excess weight in children represents a public health issue, and is associated with endothelial damage and metabolic abnormalities.^1^ ^,^ ^5^ ^,^ ^6^

The injured endothelium plays an important role in the development of many cardiovascular diseases,^7^ such as atherosclerosis and coronary heart disease.^7^ Furthermore, it may be used as a risk predictor for such events.^8^ Endothelial dysfunction is the major event in the development of atherosclerosis, and it may be observed a long time before the appearance of structural atherosclerotic disease.^9^

For the clinician, even considering that he will not make endothelial evaluation of all his patients, the knowledge of the comorbidities linked to obesity is crucial, because it is an opportunity of reinforcing the risks associated with this condition. The non-invasive evaluation of endothelial function shows the potential for cardiovascular risk stratification in children^10^ and, among such methods, main flow-mediated dilation (FMD) and pulsatility index change (PI-C) of the brachial artery stand out because they are safe, reproducible, and relatively simple techniques to be performed;^9^ which may be applied in children.^10^ ^,^ ^11^ Nevertheless, there is no previously published study assessing the variation in the pulsatility index of the brachial artery as a marker of endothelial dysfunction, comparing obese and non-obese children. Therefore, in the present study, we aimed to compare ultrasonographic, clinical and metabolic markers of cardiovascular risk between obese and eutrophic children.

## METHOD

It is a descriptive cross-sectional study with patients attending in the outpatient pediatric clinics of the primary health care public network in Ribeirão Preto, Brazil. Children aged between 6 and 10 years old were invited to participate if they were either obese [Body Mass Index (BMI) >95 percentile] or eutrophic [BMI >5th percentile and BMI <85th percentile]. The classification of children as obese or eutrophic was based on normality curves of the National Centers for Health Statistics (NCHS), considering BMI for gender and age.^12^ This reference was chosen due to its being the curve used at the service where the data was collected. No matching criterion was adopted. The only exclusion criterion adopted was failure to perform all the proposed evaluation. The sample size was estimated based on other studies with flow-mediated dilation. In a parallel-design study, 23 subjects would be required to detect an FMD difference of 60% (two-tailed) (e.g., 5 vs. 8%, at 90% power). To achieve an FMD difference of 40% (e.g., 5 vs. 7%, at 90% power), 46 subjects should be included.^13^ Considering that an absolute difference of 4 to 8% may be due to a personal variation and that a minimum of 2% difference when comparing two groups would be considered as the minimal clinically important,^13^ ^,^ ^14^ ^,^ ^15^ 40 children were evaluated in each group.

All children’s legal guardians were fully informed and had given written informed consent before the children were enrolled in this study. The study was approved by the local ethics committee.

Anthropometric parameters (weight, height, BMI), systolic and diastolic blood pressure, body fat percentage, basal metabolic rate, laboratory markers (fasting insulin, fasting glucose, quantitative index of insulin sensitivity [QUICKI], homeostasis assessment model of insulin resistance [HOMA-IR)], total cholesterol, HDL-cholesterol and LDL- cholesterol were studied. Some sonographic parameters were also evaluated (flow-mediated dilation (FMD), a variation of the pulsatility index (PI- C) and basal diameter of the brachial artery). The examinations were performed in the morning (7-9 am) after 15 minutes of rest in dorsal decubitus, in a room with temperature control (20-23ºC). All individuals were fasting for a minimum of 12 hours and had rested the night before for at least 8 hours. Initially, blood pressure was taken in the left arm with a standard mercury sphygmomanometer, establishing systolic blood pressure (SBP) and diastolic blood pressure (DBP). Weight and height were used to determine the BMI. The percentage of body fat and the basal metabolic rate were assessed by bioelectrical impedance analyzer (BIA), equipment BF-906 (Maltron^®^, UK). The sonographic evaluation used a linear probe 6-12 MHz present in the HD7 device (Philips Medical Systems^®^, Bothell, WA) attached to an electrocardiogram. All anthropometric, blood pressure and sonographic assessments were performed by a single observer.

The technique for obtaining FMD and PI-C of the brachial artery was performed as follows: after the rest period, an ultrasound image of the right brachial artery in the longitudinal plane was obtained, 5-10 cm proximal to the antecubital fossa, visualizing the intima interface and the vessel lumen in both vascular walls. A clip of 5 seconds was recorded, which would be evaluated at the end of echographic exam. The spectral Doppler mode was adjusted and standardized with a frequency of 5 MHz, 50 Hz filter and sample volume of 1.0 mm. The sample was positioned in the middle of the brachial artery at a maximum angle of 60º. Using the product software, the following variables of three consecutive similar waves were determined: peak systolic velocity (PSV), end-diastolic velocity (EDV), mean velocity (MV) and pulsatility index (PI) [PI = (PSV- DV)/MV].^16^

A pneumatic compression (standardized at 200 mmHg) was performed for 5 minutes on the right forearm just below the medial epicondyle of the children. After this period, a rapid sphygmomanometer deflation was performed. One minute after deflation, another 5-second echographic clip of the brachial artery was recorded and stored. Then, the Doppler was held between 70-80 seconds after cuff release to obtain the Doppler parameters to determine new PI values, which were recorded on the apparatus.

After examination, the values of baseline brachial artery diameter (BDpre) and 1 minute after stimulation (BDpost) were determined.^17^ For these measures, it was subjectively chosen the best image of the vessel during the end-diastolic (R wave in the electrocardiogram) to perform 3 measures, from intima proximal to intima distal.

Blood samples (20 mL) were collected and stored in conical tubes (Becton Dickinson, Plymouth, UK). The serum was stored at -80ºC for simultaneous determination of the following variables: fasting glucose, determined by the oxidase method using a Konelab 60i analyzer (WienerLab^®^, Rosario, Argentina); total cholesterol and HDL-cholesterol, determined by enzymatic method, with BT 3000BTplus analyzer (WienerLab, Rosario, Argentina), LDL cholesterol calculated according to the formula: LDL cholesterol = (Total cholesterol) - ((HDL) + (Triglycerides/5), fasting insulin, measured by chemiluminescence with DPCImmulite 2000 (DiagnosticProductsCorporation^®^, LosAngeles, CA). Insulin resistance was evaluated by checking the QUICKI; QUICKI = 1/(log [fasting glucose (mg/dl)] + log [fasting insulin (pIU/mL)]) and the homeostasis model assessment of insulin resistance (HOMA-IR), HOMA-IR = fasting glucose (mg/dL) x fasting insulin (pU / mL)/405.^18^

Data were analyzed by the SPSS program 18.0 (SPSS Inc., Chicago, USA). Descriptive statistics were performed to establish mean, standard deviation, minimum and maximum values of the studied parameters. The comparison of quantitative parameters between groups was demonstrated by unpaired t test or Mann-Whitney. The level of statistical significance was set at p<0.05.

## RESULTS

A total of 92 children were assessed for eligibility. Of those, 83 agreed to participate in the study: 41 obese children and 42 eutrophic. From those, 3 children did not complete laboratory (1 obese and 2 eutrophic) and were excluded from the final analysis. Weight, BMI, systolic and diastolic blood pressure, body fat percentage, andbasal metabolic rate were significantly different between groups. No significant difference was observed in age (Table 1).

**Table 1:** Anthropometric parameters, arterial blood pressure and body composition in obese children and controls.

	Obese (N=40)	Control (N=40)	p-value*
Age (years)	7.8±1.1	7.6±1.1	0.82
Weight (Kg)	45.9±10.45	25.4±4.4	<0.01
BMI (Kg/m^2^)	24.5±3.4	15.4±1.4	<0.01
Systolic pressure (mmHg)	97.7±8.4	89.0±5.8	<0.01
Diastolic pressure (mmHg)	64.3±7.9	52.9±5.2	<0.01
Body fat (%)	45.1±5.9	21.3±6.0	<0.01
Basal Metabolic Rate (Kcal)	1,216.1±102.1	1,072.9±66.4	<0.01

Data presented as mean ± standard deviation; *unpaired t test or Mann-Whitney U test were used depending on the distribution of the data.

When comparing the laboratory and sonographic findings of endothelial dysfunction (Table 2) in obese individuals, it was observed higher rates of total cholesterol (164.7±25.2 vs. 15.8±153.4; p=0.03), fasting insulin (7.1±5.2 vs. 2.8±1.8 pIU/mL; p<0.01), HOMA-IR (1.5±1.1 vs. 0.60±0.34; p<0.01), basal diameter of the brachial artery (2.5±0.3 vs. 2.1±0.3 mm; p<0.01). Additionally, obese children showed lower values: QUICKI (0.38±0.05 vs. 0:43±0.03, p<0.01), FMD (6.6±3.2 vs. 15.6±7.3%; p<0.01) and PI-C (-15.5±27.2 vs. -31.9±15.5%; p<0.01). There was no significant difference in fasting glucose, HDL-cholesterol and LDL-cholesterol.

**Table 2:** Ultrasonographic and laboratorial parameters in obese children and controls.

	Obese (N=40)	Control (N=40)	p-value*
Fasting insulin (pUI/mL)	7.1±5.2	2.8±1.8	<0.01
Fasting glucose (mg/dL)	82.4±5.9	83.6±6.2	0.38
QUICKI	0.38±0.05	0.43±0.03	<0.01
Total cholesterol (mg/dL)	164.7±25.2	153.4±15.8	0.03
HDL-cholesterol (mg/dL)	59.8±5.9	60.1±4.6	0.46
LDL-cholesterol (mg/dL)	83.1±22.2	77.9±13.7	0.32
HOMA-IR	1.5±1.1	0.6±0.4	p<0.01
BD pre(mm)	2.5±0.3	2.1±0.3	p<0.01
FMD (%)	6.6±3.2	15.6±7.3	p<0.01
PI-C (%)	-15.5±27.2	-31.9±15.5	p<0.01

Data presented as: mean ± standard deviation; QUICKI: quantitative insulin sensitivity check index; HOMA-IR: homeostasis model of assessment - insulin resistance; BD pre: basal diameter of the brachial artery; FMD: brachial artery flow mediated dilation; PI-C: pulsatility index change. *unpaired t test or Mann-Whitney U test were used depending on the distribution of the data.

## DISCUSSION

The present study aimed to improve the evaluation of the endothelial dysfunction in obese children through a noninvasive method, compared to already acknowledged clinical, laboratory and metabolic markers of cardiovascular risks.^19^ ^,^ ^20^ It is an opportunity to introduce the study of the PI-C of the brachial artery, method with good reliability and greater technical facility at sonographic analysis of endothelial dysfunction when compared to FMD.^21^

Regarding metabolic parameters, significant increased values of systolic and diastolic pressure, body fat, and basal metabolic rate among obese children were observed. There was evidence of worsening of insulin resistance markers with significantly increased fasting insulin and HOMA-IR; and decreased QUICKI in the obese group, compared to the eutrophic group. Regarding the study of endothelial function, it was observed significantly lower values of FMD and PI-C in the obese group.

Some previous studies have shown a correlation between obesity in children and changes in laboratory markers, especially total cholesterol,^22^ LDL-cholesterol, HDL-cholesterol, triglycerides, fasting glucose and basal insulin^20^ ^,^ ^23^ ^,^ ^24^ ^,^ ^25^ partially coincident with some results found in the present study. Furthermore, the positive correlation between HOMA-IR with metabolic syndrome, BMI, waist circumference and weight in children was already described;^26^ as well as lower QUICKI scores in obese adult individuals.^26^ ^,^ ^27^ Those markers showed significant differences in the investigated groups of children, which translates the increased insulin resistance in the obese.

When examining the results of the present study regarding endothelial function, significantly decreased FMD-C and PI in obese children were observed. The main findings of noninvasive markers of cardiovascular risks studied are: decreased FMD of the brachial artery and increased intima medial thickness (IMT) of the carotid artery, both in children^28^ ^,^ ^29^ ^,^ ^30^ ^,^ ^31^ ^,^ ^32^ and adults.^23^ ^,^ ^27^ ^,^ ^33^ The evaluation of the variation of PI-C of the brachial artery 1 minute after compression of the forearm for 5 minutes was evaluated for the first time as an endothelial marker for children in this study. Among adults, high correlations to age, BMI, waist circumference, cholesterol, systolic blood pressure, and IMT were already demonstrated.^27^ Moreover, the technique appears to be easier to learn and it has a good reproducibility when compared to FMD.^21^ The contribution of the present study for clinicians is not the suggestion of evaluating endothelium of all his patients, but the possibility of doing it and the knowledge that the comorbidities linked to obesity are frequent and have an early effect on the cardiovascular system.

The main limitation of the study was the selection bias. Cross sectional studies, using convenient and not randomized samples add a selection bias that could only be eliminated by conducting a cohort study. Another important limitation refers to the inclusion of scholar children without considering their pubertal stage and gender. During this period, the variability of maturation and the differences between boys and girls, may influence the results, because some children under the age of 10, mainly when obesity is present, already started puberty. On the other hand, considering the innovative aspect of the sonographic evaluation, the results presented, even considering the insufficient external and internal validity, may be useful to launch the discussion about the importance of evaluating, through medical images, the evolution of the atherosclerotic process during childhood. Studies of biomarkers and non-invasive markers of endothelial dysfunction have increased in recent years in the search for the best method of clinical applicability as a tool for research in overweight and obese children.^34^ Hence, in view of the changing lifestyle and increasing childhood obesity, there is a continuing need to advance the prevention and early diagnosis of cardiovascular and metabolic alterations imposed by this pathology, in order to modify the natural history of the disease.^35^

In conclusion, obesity worsens ultrasonographical and laboratorial markers of metabolic and cardiovascular disease risk in children.
